# Outcomes of Patients Discharged From a First Episode Psychosis Service in Derby City and South County

**DOI:** 10.1192/bjo.2025.10511

**Published:** 2025-06-20

**Authors:** Mahendra Kumar, Remon Mosaad

**Affiliations:** Derbyshire Healthcare Foundation Trust, Derby, United Kingdom

## Abstract

**Aims:** The Early Intervention for Psychosis (EIP) service in Derby City and Derbyshire South County provides care for individuals aged 14–65 experiencing a first episode of psychosis. The service supports a diverse population across Derby City (Census 2021 population: 261,400) and Derbyshire South County (Census 2021 population: 349,000), reflecting varying demographic and clinical characteristics. This study examines diagnostic outcomes, referral sources, and discharge destinations of discharged patients.

Aim was to ascertain the diagnostic outcomes, referral sources, and discharge destinations of patients discharged from the EIP service in Derby City and Derbyshire South County.

**Methods:** All patients discharged from the EIP service between 1 April 2023 and 1 April 2024 were included. Included patients were under the service for at least 3 months. Some continued up to 3 years, while others were discharged earlier for reasons such as non-psychotic diagnoses. Data on diagnosis, referral source, and discharge destination were retrospectively collected from clinical records, recorded in an Excel spreadsheet, and analysed to identify key patterns and trends.

**Results:** Nearly half of discharged patients (46.67%) had a psychosis spectrum diagnosis (F20–F29; ICD-10). Organic psychoses (4.4%), drug-induced psychosis (8.8%), bipolar disorder with psychotic symptoms (11.1%), other mood-related psychoses (6.6%), and non-psychotic conditions (22.2%) were also identified.

Referrals came primarily from secondary mental health services (48.89%), inpatient units (34.4%), primary care (12.2%), and the Court Liaison and Diversion Service (4.4%).

Discharge destinations showed that 42.7% of patients were transferred to Community Mental Health Teams, and 47.1% were discharged to primary care. Smaller proportions were discharged to learning disabilities services (1.1%), out-of-area early intervention for psychosis services (7.87%), or the perinatal team (1.1%).

**Conclusion:** The Derby EIP caseload aligns with the service’s focus on first episode psychosis. Low referral rates from primary care indicate that many patients are first identified in crisis settings. However, the majority of patients being discharged to primary care highlights the effectiveness of an intensive, multidisciplinary approach. The small number of referrals to specialized services reinforces positive outcomes in EIP patients.

